# Texture-Based MRI Analysis Reveals Microstructural Alterations in the Putamen in Bipolar Disorder

**DOI:** 10.3390/medicina62050914

**Published:** 2026-05-08

**Authors:** Özlem Gül, Sema Baykara, Mustafa Nuray Namlı, Murat Baykara

**Affiliations:** 1 Department of Psychiatry, Faculty of Medicine, Istinye University, 34396 Istanbul, Türkiye; 2 Department of Psychiatry, Erenkoy Psychiatry and Neurology Training and Research Hospital, 34738 Istanbul, Türkiye; semabaykara@hotmail.com; 3 Department of Psychiatry, Bakirkoy Prof Mazhar Osman Training and Research Hospital for Psychiatry, Neurology, and Neurosurgery, 34147 Istanbul, Türkiye; mnnamli@gmail.com; 4 Department of Radiology, Haydarpasa Numune Training and Research Hospital, 34668 Istanbul, Türkiye; muratbaykara@hotmail.com

**Keywords:** bipolar disorder, putamen, magnetic resonance imaging, texture analysis, neuroimaging, brain structure, image processing, radiomics

## Abstract

*Background and Objectives*: Bipolar disorder (BD) is associated with widespread neuroanatomical alterations, particularly within subcortical structures involved in emotional regulation. Conventional magnetic resonance imaging (MRI) approaches may fail to detect subtle microstructural changes. This study aimed to evaluate histogram-based texture characteristics of the putamen in patients with BD and to compare these findings with those of healthy controls. *Materials and Methods*: This retrospective cross-sectional study included 66 participants (33 BD patients, 33 controls). All subjects underwent standardized cranial MRI. Regions of interest corresponding to the putamen were manually delineated, and histogram-based texture parameters were extracted using custom-developed software. Group comparisons were performed using appropriate statistical tests based on data distribution. *Results*: The groups were comparable in age and sex (*p* > 0.05). Significant differences were observed in multiple texture parameters, particularly in the left putamen. Mean and median values were significantly higher in BD patients compared to controls (511.19 ± 106.96 vs. 440.68 ± 102.21, *p* = 0.008; 511.92 ± 106.71 vs. 440.53 ± 102.74, *p* = 0.007). Minimum intensity values and root-sum-of-squares levels were also significantly increased (*p* < 0.001). Skewness differed significantly (*p* = 0.004), indicating altered distribution asymmetry. Percentile analyses demonstrated consistent differences across nearly all levels, suggesting a shift in intensity distribution. Additionally, Katz fractal dimension was significantly lower in BD patients (*p* < 0.001), indicating reduced structural complexity. Similar but less pronounced alterations were observed in the right putamen. Overall, the findings suggest the presence of widespread alterations in intensity distribution and structural characteristics. *Conclusions*: Patients with BD exhibit significant alterations in putamen texture parameters, potentially reflecting alterations in intensity distribution and texture-derived structural characteristics. Histogram-based texture analysis may provide a sensitive, non-invasive approach for detecting subtle brain alterations in BD and may serve as a complementary neuroimaging biomarker.

## 1. Introduction

Bipolar disorder (BD) is a chronic, recurrent psychiatric disorder characterized by alternating episodes of mania, hypomania, and depression, and is associated with substantial functional impairment and reduced quality of life. The disorder has a complex and multifactorial pathophysiology involving genetic, neurochemical, and neuroanatomical components. In recent decades, neuroimaging studies have provided important insights into the biological underpinnings of BD, demonstrating that the disorder is associated with widespread structural and functional alterations in brain regions involved in emotional regulation, cognitive control, and reward processing [1,2]. These findings support the view that BD represents a disorder of distributed neural networks rather than a dysfunction confined to a single brain region. The clinical course of bipolar disorder is highly heterogeneous, with variable treatment responses and symptom trajectories across different phases of illness, highlighting the need for objective biomarkers to guide personalized management [3].

Among the neural systems implicated in BD, the fronto-limbic and fronto-striatal circuits have received particular attention. These networks integrate cortical and subcortical structures, including the prefrontal cortex, amygdala, thalamus, and basal ganglia, and play a central role in mood regulation and emotional processing. Within this framework, the basal ganglia—especially the putamen—are of considerable interest due to their involvement in motor, cognitive, and affective functions. Structural magnetic resonance imaging (MRI) studies have reported volumetric and morphological alterations in subcortical regions such as the putamen, caudate nucleus, and thalamus in patients with BD, suggesting that these structures may contribute to the pathophysiology of the disorder [4,5]. Moreover, alterations in the putamen have been linked to dysfunctions in reward processing and motivational regulation, which are core features of bipolar disorder.

Importantly, neuroanatomical abnormalities in BD are not limited to individuals with established disease but have also been observed in populations at risk, including unaffected relatives and individuals in early stages of illness. These findings suggest that structural brain alterations may represent trait-related markers or endophenotypes associated with vulnerability to BD rather than solely reflecting disease progression [6,7]. In this context, the identification of subtle and early brain changes is of particular importance for understanding disease mechanisms and for developing potential biomarkers for early diagnosis and risk stratification.

In addition to gross volumetric changes, accumulating evidence indicates that BD is associated with more subtle microstructural and signal-level alterations that may not be fully captured by conventional region-based or volumetric MRI analyses. For example, qualitative and quantitative MRI studies have demonstrated abnormalities in signal intensity distribution, white matter integrity, and tissue composition in patients with BD [6]. Similarly, studies in pediatric and early-onset BD populations have highlighted structural alterations in subcortical regions, including the putamen, suggesting that these abnormalities may emerge early in the course of the disorder and potentially precede clinical manifestations [8,9]. These findings underscore the need for more sensitive imaging approaches capable of detecting subtle and spatially distributed alterations in brain tissue.

Conventional neuroimaging approaches, which primarily rely on measurements of regional volume or mean signal intensity, may be insufficient to capture complex patterns of tissue heterogeneity and microstructural organization. In this regard, advanced quantitative imaging techniques such as histogram-based texture analysis have emerged as promising tools for the characterization of tissue properties. Texture analysis allows for the assessment of spatial variations in pixel intensity distributions and provides a range of quantitative parameters reflecting heterogeneity, distribution shape, and structural complexity. These methods have been increasingly applied in neurological and psychiatric research to identify imaging biomarkers that are not detectable using traditional metrics.

Texture-based approaches are particularly relevant in the context of BD, where microstructural alterations may manifest as subtle changes in intensity distribution rather than overt volumetric differences. By capturing global distribution shifts, asymmetry patterns, and higher-order statistical features, texture analysis may offer additional insights into disease-related brain alterations. Furthermore, given the potential lateralization of brain abnormalities in BD, evaluating hemispheric differences in texture parameters may provide important information regarding the spatial organization of these alterations. Histogram-based texture analysis provides quantitative descriptors of signal intensity distribution and heterogeneity, which may reflect underlying microstructural organization. However, these measures represent indirect imaging biomarkers and do not correspond to direct histopathological findings.

Despite the growing interest in advanced imaging techniques, the application of histogram-based texture analysis to subcortical structures such as the putamen in BD remains limited. In particular, there is a lack of studies systematically examining the distributional and complexity-related characteristics of putamen signal intensity and their potential alterations in BD. Addressing this gap may contribute to a more comprehensive understanding of the neurobiological mechanisms underlying the disorder.

Therefore, the present study aimed to evaluate histogram-based texture parameters derived from the putamen in patients with bipolar disorder and to compare these parameters with those of healthy controls. In addition, this study sought to investigate whether these texture-derived features reflect microstructural alterations and whether such changes differ between hemispheres. By applying a quantitative and distribution-sensitive imaging approach, this study aims to provide novel insights into the structural characteristics of the putamen in BD and to explore the potential of texture analysis as a complementary neuroimaging biomarker.

## 2. Materials and Methods

### 2.1. Study Design and Setting

This retrospective, cross-sectional study was conducted at a tertiary-level Training and Research Hospital specializing in psychiatric disorders. Clinical and radiological data were obtained from patients followed at the Community Mental Health Center affiliated with Bakirkoy Prof. Mazhar Osman Training and Research Hospital for Psychiatry, Neurology, and Neurosurgery, Istanbul, Türkiye. Data were collected from the records between January 2018 and July 2022.

The study protocol was approved by the Ethics Committee of University of Health Sciences, Kanuni Sultan Suleyman Training and Research Hospital, Istanbul, Türkiye (protocol code: 2022.07.175, date of approval: 7 July 2022), and all procedures were carried out in accordance with the Declaration of Helsinki. Due to the retrospective design and the use of anonymized data, the requirement for informed consent was waived.

### 2.2. Study Population

#### 2.2.1. Bipolar Disorder Group

Patients diagnosed with BD according to the Diagnostic and Statistical Manual of Mental Disorders, Fifth Edition (DSM-5), were eligible for inclusion. All patients included in the bipolar disorder group were diagnosed with Bipolar Disorder Type I according to DSM-5 criteria. Only individuals who had undergone cranial MRI for clinical indications as part of their neurological evaluation were included.

Clinical and imaging data were retrieved from the Hospital Information System (HIS). All MRI scans were reviewed by an experienced radiologist to ensure adequate image quality and anatomical suitability for analysis.

#### 2.2.2. Inclusion and Exclusion Criteria

Participants were included if they were between 18 and 65 years of age, had a diagnosis of bipolar disorder based on DSM-5 criteria, and had available and suitable cranial MRI data. Participants were excluded if they had any additional psychiatric disorder, a history of intellectual disability, known neurological or systemic diseases affecting brain structure, or a history of alcohol or substance use within the previous six months, to ensure a clinically homogeneous bipolar disorder sample.

#### 2.2.3. Control Group

The control group consisted of age- and sex-matched individuals who presented to the neurology clinic with non-specific neurological symptoms (e.g., headache) and underwent cranial MRI as part of their clinical evaluation. All included participants had no identifiable pathology on imaging and no documented psychiatric diagnosis.

### 2.3. MRI Acquisition Protocol

All MRI examinations were performed using the same imaging system to ensure methodological consistency. Imaging was conducted using a 1.5 Tesla Brivo MR355 scanner (General Electric Medical Systems, Milwaukee, WI, USA) equipped with an 8-channel head coil.

T2-weighted Fast Spin Echo (FSE) sequences were acquired under standardized imaging conditions. These sequences were selected due to their suitability for evaluating subcortical structures and tissue contrast characteristics.

### 2.4. Image Processing and Texture Analysis

MRI data were exported in Digital Imaging and Communications in Medicine (DICOM) format and transferred to a Windows 10-based workstation (Microsoft Corporation, Seattle, WA, USA) for further processing.

Image analysis was performed using a custom-developed software implemented in MATLAB (version R2021b; MathWorks, Natick, MA, USA), enabling standardized extraction of histogram-based texture features [10,11,12].

For each subject, regions of interest (ROIs) corresponding to the putamen were manually delineated on axial slices that best represented the anatomical boundaries. ROI placement was carefully performed to avoid inclusion of adjacent tissues and was conducted by an experienced senior radiologist. All image evaluations, including slice selection and ROI delineation, were performed by an experienced radiologist who was blinded to the group allocation.

Slice selection was based on consistent anatomical landmarks to ensure comparable visualization of the putamen across subjects. Due to the retrospective nature of the study and the use of routine clinical imaging, standardized stereotactic coordinates (e.g., MNI space) were not available.

Histogram-based texture parameters were subsequently extracted from the defined ROIs. These parameters characterize the distribution and variability of pixel intensities and have been widely used to assess tissue heterogeneity and microstructural alterations.

The definitions and radiological interpretations of the selected texture parameters included in the final analysis are summarized in Table 1.

### 2.5. Outcome Measures

The primary outcome was the comparison of histogram-based texture parameters of the putamen between patients with BD and healthy controls. These parameters were considered indirect markers of potential microstructural brain alterations associated with BD.

### 2.6. Handling of Missing Data

All variables were assessed for completeness prior to analysis. Cases with missing or inadequate MRI data, poor image quality, or incomplete clinical information were excluded during the preprocessing stage. Therefore, only complete cases were included in the final analysis, and no imputation methods were applied.

### 2.7. Bias and Confounding Control

Several strategies were implemented to minimize potential bias. Selection bias was reduced by applying predefined inclusion and exclusion criteria consistently across both groups. Measurement bias was minimized through the use of standardized MRI acquisition protocols and ROI delineation by an experienced radiologist.

To control for confounding, the control group was matched to the BD group based on age and sex. Additionally, identical exclusion criteria were applied to both groups to enhance internal validity.

### 2.8. Sample Size Considerations

All eligible patients meeting the inclusion criteria during the study period were included. Due to the retrospective nature of the study, no a priori sample size calculation was performed.

### 2.9. Statistical Analysis

All statistical analyses were performed using Wistats v3.0 (WisdomEra Corp., Istanbul, Turkey), a Python-based analytical platform integrating SciPy, scikit-learn, and statsmodels libraries (https://wistats.wisdomera.io, accessed on 27 March 2026).

The distribution of continuous variables was evaluated using skewness, kurtosis, and the Shapiro–Wilk test. Depending on distribution characteristics, continuous variables were compared using the independent samples *t*-test or the Mann–Whitney U test. Categorical variables were analyzed using the Chi-square test or Fisher’s exact test, as appropriate.

All statistical tests were two-sided, and a *p*-value of <0.05 was considered statistically significant.

## 3. Results

### 3.1. Baseline Characteristics

A total of 66 participants were included in the final analysis, comprising 33 patients with BD and 33 healthy controls. The two groups were comparable in terms of demographic characteristics. The proportion of female participants was 57.6% in the BD group and 54.5% in the control group (*p* = 0.500). The mean age was 36.42 ± 12.69 years in the BD group and 37.42 ± 13.64 years in controls, with no statistically significant difference (*p* = 0.807), indicating appropriate matching between groups (Table 2).

### 3.2. Left Putamen Texture Analysis

Significant differences were observed in multiple texture parameters of the left putamen between BD patients and controls. Mean intensity and median values were significantly higher in the BD group (511.19 ± 106.96 vs. 440.68 ± 102.21, *p* = 0.008; and 511.92 ± 106.71 vs. 440.53 ± 102.74, *p* = 0.007, respectively). Similarly, minimum intensity values were markedly increased in BD patients (*p* < 0.001) (Table 2).

Additionally, the most frequent value and root-mean-square level were significantly higher in the BD group (*p* = 0.007 and *p* = 0.009, respectively), along with a substantial increase in root-sum-of-squares level (*p* < 0.001). Skewness values also differed significantly between groups (*p* = 0.004), suggesting alterations in signal distribution asymmetry.

Percentile-based analyses demonstrated consistent and statistically significant differences across nearly all percentile levels, including the 10th through 90th percentiles (*p*-values ranging from 0.005 to 0.016), suggesting a potential shift in intensity distribution rather than isolated changes.

**Figure 1:**
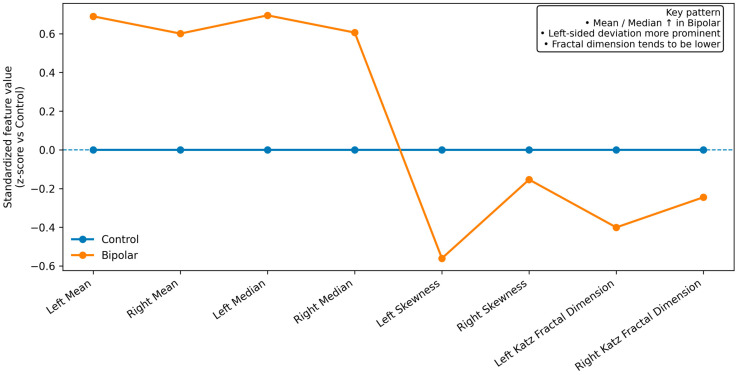
The texture signature profile of putamen features. Standardized (z-score) comparison of selected texture parameters between bipolar disorder and control groups. Bipolar patients show higher mean and median values, altered skewness, and reduced fractal dimension, with more pronounced deviations in the left putamen.

Moreover, the Katz fractal dimension was significantly lower in the BD group (*p* < 0.001), suggesting alterations in structural complexity. These feature-level differences, including increased central tendency measures and reduced structural complexity, are visually summarized in Figure 1.

### 3.3. Right Putamen Texture Analysis

The right putamen analysis also revealed significant differences, although less pronounced compared to the left side. Mean and median intensity values were significantly higher in BD patients (*p* = 0.003 for both). Minimum and maximum intensity values were also significantly increased (*p* = 0.002 and *p* = 0.016, respectively) (Table 2).

Similar to the left side, the most frequent value and root-mean-square level were significantly higher in BD patients (*p* = 0.001 and *p* = 0.003, respectively), along with a marked increase in root-sum-of-squares level (*p* < 0.001).

**Figure 2:**
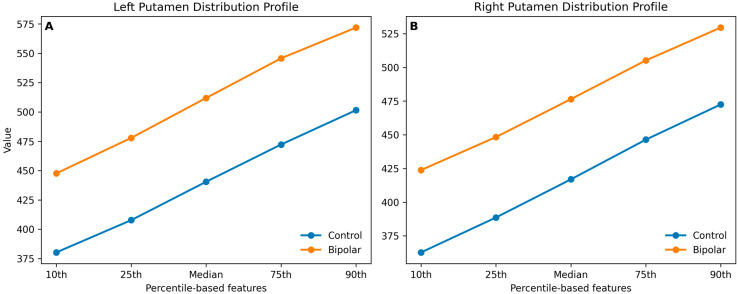
The distribution shift across percentile-based texture features. Comparison of percentile-derived intensity parameters in the left (**A**) and right (**B**) putamen between bipolar disorder and control groups. Bipolar patients demonstrate a consistent upward shift across the distribution, indicating global intensity alteration, with stronger effects observed in the left hemisphere.

Percentile analyses again showed widespread differences across distribution metrics, with significant differences observed from the 10th to the 90th percentiles (*p*-values ranging from 0.002 to 0.008), supporting the presence of a systematic alteration in signal intensity distribution. The global distribution shift across percentile-based parameters is illustrated in Figure 2.

### 3.4. Overall Findings

Overall, patients with BD exhibited widespread and statistically significant differences in putamen texture parameters compared to healthy controls. These differences were more pronounced in the left putamen. The findings indicate alterations in intensity distribution and texture-derived characteristics that may reflect underlying structural differences detectable through histogram-based analysis.

## 4. Discussion

In this study, we aimed to evaluate histogram-based texture characteristics of the putamen in patients with bipolar disorder and to compare these findings with those of healthy controls. The main findings can be summarized as follows: (i) patients with BD exhibited significantly higher intensity-based parameters in both putamina, particularly in the left hemisphere; (ii) there was a consistent global shift in intensity distribution across multiple percentile levels; (iii) higher-order features such as skewness and fractal dimension indicated altered signal asymmetry and reduced structural complexity; and (iv) these alterations appeared to be more pronounced in the left putamen. Specifically, mean and median values were significantly higher in the left putamen of BD patients (511.19 ± 106.96 vs. 440.68 ± 102.21, *p* = 0.008; 511.92 ± 106.71 vs. 440.53 ± 102.74, *p* = 0.007), along with marked differences in minimum values (*p* < 0.001) and root-sum-of-squares levels (*p* < 0.001). These findings collectively indicate that BD is associated with differences in intensity distribution and texture-derived features.

The involvement of the putamen in BD observed in this study is consistent with previous neuroimaging research. Structural MRI studies have repeatedly demonstrated abnormalities in subcortical regions, including the putamen, in patients with BD [4,5]. Furthermore, alterations in putamen volume and morphology have been reported not only in patients but also in individuals at risk, suggesting that these changes may represent vulnerability markers [6,7]. Our findings extend this knowledge by demonstrating that, beyond volumetric differences, there are also significant alterations in intensity distribution and structural complexity within the putamen. Previous studies have also reported structural and shape alterations in the basal ganglia, including the putamen, in patients with bipolar disorder [13]. Meta-analytic evidence further supports the presence of gray matter alterations in bipolar disorder across multiple brain regions, including subcortical structures [14]. Radiomics and texture-based approaches have been increasingly recognized as powerful tools for capturing tissue heterogeneity beyond conventional imaging metrics, supporting their potential role in identifying subtle imaging biomarkers [15,16].

Dopaminergic dysfunction within the striatum, particularly involving the putamen, has also been implicated in the pathophysiology of bipolar disorder [17]. Increased dopaminergic transmission during manic episodes and reduced dopamine transporter density in the putamen have been associated with elevated synaptic dopamine levels and greater symptom severity. Although the present study does not directly assess neurotransmitter systems, the observed alterations in texture-derived parameters may reflect underlying microstructural changes related to such neurobiological processes. In addition, experimental studies using animal models have provided further evidence supporting the role of dopaminergic dysregulation in bipolar disorder [18]. Alterations in dopamine signaling have been associated with both behavioral changes and structural modifications within striatal regions. These findings offer a translational perspective and may help contextualize the observed texture-based alterations, although direct mechanistic links cannot be established within the scope of the present study.

One of the most important contributions of this study is the demonstration of a potential shift in voxel intensity distribution across a wide range of percentile levels. Unlike conventional approaches that focus on mean or regional volume, our results show that the entire distribution—from lower to higher percentiles—is altered in BD. This finding suggests that microstructural changes are not localized but rather diffuse and systematic. Similar observations have been reported in texture analysis studies of neurological diseases, where subtle signal heterogeneity and distribution changes were detected even in the absence of visible structural abnormalities [19,20].

Higher-order texture features further support the presence of microstructural alterations. In our study, skewness differed significantly in the left putamen (*p* = 0.004), indicating asymmetry in signal intensity distribution. Additionally, the Katz fractal dimension was significantly lower in BD patients (*p* < 0.001), suggesting reduced structural complexity. Fractal analysis has been proposed as a sensitive method for capturing subtle alterations in brain organization that are not detectable using traditional volumetric measures [21,22]. Our findings are consistent with previous reports indicating that disease-related processes may lead to reduced complexity and altered spatial organization of brain tissue.

The observed pattern, with more pronounced alterations in the left putamen, may suggest a degree of hemispheric asymmetry. However, as no direct statistical comparison between hemispheres was performed, this observation should be interpreted cautiously. Previous studies have suggested that BD may be associated with lateralized brain abnormalities, particularly within fronto-striatal circuits [1]. Although volumetric studies have produced heterogeneous results, the present findings may indicate that texture-based measures could be sensitive to subtle asymmetrical patterns, which warrants further investigation.

From a methodological perspective, the use of histogram-based texture analysis provides a more comprehensive characterization of tissue properties compared to traditional imaging metrics. Texture analysis allows for the quantification of heterogeneity, distribution shape, and signal complexity, thereby offering additional information beyond mean intensity or volume. Previous studies have emphasized the potential of radiomics and texture-based approaches to identify imaging biomarkers in neurological and psychiatric conditions, although challenges related to reproducibility and standardization remain [23,24]. Our findings support the growing body of evidence suggesting that advanced quantitative imaging techniques can reveal clinically relevant microstructural alterations. Texture-derived parameters are thought to reflect underlying tissue characteristics such as heterogeneity, spatial organization, and signal complexity. These features may be influenced by factors including cellular density, microstructural organization, and tissue composition. However, as these measures are derived from signal intensity patterns, they should be interpreted as indirect indicators rather than direct representations of histological structure.

Despite these strengths, several limitations should be acknowledged. First, the relatively small sample size may limit the generalizability of the findings and reduce statistical power, increasing the risk of both type I and type II errors. Second, the retrospective design introduces the possibility of selection bias, although predefined inclusion and exclusion criteria were applied to minimize this effect. ROI delineation was performed manually, which may introduce observer-related variability, despite being conducted by an experienced radiologist. In addition, MRI acquisition was performed using a single scanner and protocol, which improves internal consistency but may limit external reproducibility. The use of axial slices instead of a volumetric approach represents a methodological limitation. However, axial T2-weighted images provided optimal visualization of putamen boundaries in routine clinical practice and enabled consistent extraction of texture features. In addition, variability in acquisition parameters and slice thickness in retrospective datasets limited the feasibility of standardized volumetric analysis.

Although the putamen contains anatomically and functionally distinct subregions, subregional segmentation was not performed in the present study. On conventional MRI, the putamen appears relatively homogeneous on visual inspection, which limits the reliability of subregional delineation in routine clinical images. Therefore, texture analysis was applied to the entire putamen to capture global distributional characteristics. Future studies using high-resolution imaging and atlas-based segmentation approaches may help to further elucidate subregional differences.

A significant difference in ROI pixel count between groups was observed, which may have influenced intensity-based texture parameters. Since several histogram-derived features are sensitive to ROI size, this represents a potential confounding factor and should be considered when interpreting the results. Furthermore, given the large number of statistical comparisons performed, the risk of type I error cannot be excluded, as no formal correction for multiple testing was applied.

Clinical variables such as medication status, illness duration, and mood state were not controlled, which may have influenced the observed imaging differences. Finally, although histogram-based texture parameters provide indirect information about tissue characteristics, they do not allow for direct histopathological validation, and therefore, their biological interpretation should be made with caution.

Future research should aim to validate these findings in larger, multicenter cohorts and to integrate histogram-based texture features with other imaging modalities, such as diffusion and functional MRI. Additionally, the application of normalization strategies or volume-adjusted analyses may help mitigate potential ROI size-related effects observed in the present study. The incorporation of machine learning approaches may further enhance the identification of clinically relevant imaging biomarkers and improve diagnostic accuracy [25,26]. Longitudinal designs will also be essential to determine whether these imaging features are associated with disease progression, treatment response, or clinical outcomes.

## 5. Conclusions

In conclusion, this study demonstrates that patients with bipolar disorder exhibit significant alterations in putamen texture parameters, potentially reflecting global intensity shifts and reduced microstructural complexity. These changes were more pronounced in the left putamen. Histogram-based texture analysis appears to be a promising tool for detecting subtle brain alterations that are not captured by conventional imaging methods. These findings contribute to a better understanding of the neurobiological basis of BD and highlight the potential role of advanced quantitative imaging techniques as complementary biomarkers in psychiatric research.

## Figures and Tables

**Table 1:** Definitions of Histogram-Based and Texture Analysis Parameters Used for Putamen Evaluation.

Parameter	Definition	Interpretation in MRI Texture Analysis
Pixel Count of ROI	Total number of pixels within the selected ROI	Represents the 2D area of the segmented putamen (not volumetric)
Mean	Average intensity value of pixels within ROI	Reflects overall signal intensity of the tissue
Median	Middle value of intensity distribution	Robust measure of central tendency, less affected by outliers
Minimum	Lowest intensity value in ROI	Indicates the lowest signal component within the tissue
Maximum	Highest intensity value in ROI	Indicates the highest signal component within the tissue
Most Frequent Value	Most common intensity value within ROI	Represents the dominant tissue signal intensity
Skewness	Measure of asymmetry of intensity distribution	Indicates shift toward higher or lower intensity values
Root-Mean-Square Level	Quadratic mean of intensity values	Reflects overall signal magnitude and energy distribution
Root-Sum-of-Squares Level	Sum of squared intensity values	Represents total signal energy within the ROI
Percentiles (10th, 25th, 75th, 90th)	Intensity thresholds at specified distribution levels	Provide information on global distribution shifts and tissue heterogeneity
Katz Fractal Dimension	Measure of structural complexity	Higher values indicate more complex and irregular tissue patterns

ROI: Regions of Interest.

**Table 2:** Comparative Outcomes Between Bipolar and Control Groups According to Putamen Texture Analysis.

	Bipolar Mean ± SD, *N* (%)	Control Mean ± SD, *N* (%)	*p*
*N*	33 (50%)	33 (50%)	
Gender			0.500
Female	19 (57.6%)	18 (54.5%)	
Male	14 (42.4%)	15 (45.5%)	
Age	36.42 ± 12.69	37.42 ± 13.64	0.807
Left Putamen			
Left Pixel Count of ROI	948.12 ± 186.43	790.48 ± 287.85	<0.001
Left Mean	511.19 ± 106.96	440.68 ± 102.21	0.008
Left Median	511.92 ± 106.71	440.53 ± 102.74	0.007
Left Minimum	372.97 ± 82.70	306.36 ± 70.98	<0.001
Left Maximum	645.82 ± 152.70	587.55 ± 147.00	0.113
Left Most Frequent Value	506.39 ± 108.06	435.61 ± 99.33	0.007
Left Skewness	−0.11 ± 0.23	0.11 ± 0.39	0.004
Left Root-Mean-Square Level	513.58 ± 107.82	443.34 ± 103.47	0.009
Left Root-Sum-of-Squares Level	15,623.62 ± 3256.80	12,191.44 ± 3110.81	<0.001
Left 10th Percentile	447.64 ± 91.89	380.29 ± 81.80	0.005
Left 25th Percentile	477.92 ± 97.50	407.80 ± 90.24	0.005
Left 75th Percentile	545.70 ± 117.31	472.27 ± 114.69	0.012
Left 90th Percentile	571.89 ± 124.61	501.51 ± 124.34	0.016
Left Katz Fractal Dimension	1.23 ± 0.05	1.25 ± 0.05	<0.001
Right Putamen			
Right Pixel Count of ROI	1083.97 ± 204.21	800.06 ± 253.24	<0.001
Right Mean	476.33 ± 102.04	417.19 ± 98.46	0.003
Right Median	476.47 ± 101.68	416.95 ± 98.24	0.003
Right Minimum	344.94 ± 79.87	288.76 ± 72.02	0.002
Right Maximum	590.76 ± 128.25	538.21 ± 139.55	0.016
Right Most Frequent Value	476.06 ± 102.98	407.76 ± 88.04	0.001
Right Skewness	−0.09 ± 0.23	−0.05 ± 0.28	0.500
Right Root-Mean-Square Level	478.17 ± 102.39	419.47 ± 99.38	0.003
Right Root-Sum-of-Squares Level	15,678.71 ± 3914.41	11,694.06 ± 3259.30	<0.001
Right 10th Percentile	423.84 ± 92.23	362.74 ± 82.60	0.002
Right 25th Percentile	448.33 ± 96.44	388.64 ± 89.28	0.002
Right 75th Percentile	505.14 ± 108.21	446.41 ± 106.89	0.005
Right 90th Percentile	529.50 ± 114.28	472.50 ± 117.31	0.008
Right Katz Fractal Dimension	1.25 ± 0.06	1.27 ± 0.07	0.327

Comparison of histogram-based texture parameters derived from the putamen between patients with bipolar disorder and healthy controls. Clinically relevant parameters are presented to enhance interpretability. The results demonstrate a consistent shift in intensity distribution and structural complexity, particularly in the left putamen. ROI: Regions of Interest; *N*: number of cases.

## Data Availability

The data generated and analyzed in this study are available through the Istinye University Dataset Sharing Platform. De-identified clinical data can be accessed at the following link: https://dataset.istinye.edu.tr/dataset?did=92 (accessed on 28 March 2026). All data were fully anonymized in accordance with applicable ethical and data protection regulations. Access to the dataset is restricted to research purposes within a controlled-access framework, in line with the platform’s data-sharing and licensing policies. The shared dataset includes processed, de-identified variables suitable for analysis, rather than raw clinical data, to ensure compliance with ethical and privacy standards.

## Abbreviations

The following abbreviations are used in this manuscript:

BD	Bipolar Disorder
MRI	Magnetic Resonance Imaging
ROI	Region of Interest
DSM-5	Diagnostic and Statistical Manual of Mental Disorders, Fifth Edition
HIS	Hospital Information System
DICOM	Digital Imaging and Communications in Medicine
FSE	Fast Spin Echo
IRB	Institutional Review Board
SD	Standard Deviation
